# Prevalence of Sickle Cell Trait in the Southern Suburb of Beirut, Lebanon

**DOI:** 10.4084/MJHID.2016.015

**Published:** 2016-02-20

**Authors:** Abdel Badih El Ariss, Mohamad Younes, Jad Matar, Zeina Berjaoui

**Affiliations:** 1 Faculty of Medicine. Balamand University, Beirut, Lebanon; 2 Faculty of Sciences. Haigazian University, Beirut, Lebanon; 3 Faculty of Sciences. American University of Beirut, Beirut, Lebanon; 4 Faculty of Health Sciences – American University of Science and Technology, Beirut, Lebanon

## Abstract

**Objective:**

The objective of this study was to assess the prevalence, gender differences, and time trends of Sickle Cell Trait in the Southern Suburb of Beirut, Lebanon, as well as to highlight the importance of screening for Sickle Cell Trait carriers in this population. Another objective was to describe a new screening technique for Sickle Cell Trait carriers.

**Methods:**

This was a retrospective cohort study carried out at a private laboratory in the Southern Suburb of Beirut, Lebanon between 2002 and 2014. The sickling test was carried out for each patient using two methods: the classical “sodium metabisulfite sickling test”, and the new “sickling test method” used in the private lab. As a confirmatory test, hemoglobin electrophoresis was run on a random sample of 223 cases which were found to be positive using the two sickling tests.

**Results:**

A total of 899 cases were found to be positive for the sickle cell trait out of 184,105 subjects screened during the 12-year period, prevalence = 0.49% (95% CI: 0.46 – 0.52). Among the total sample, females were found to have higher prevalence, where no time trend over the studied period was noted. The haemoglobin electrophoresis method confirmed the results of this new sickling test technique among the random sample of the 223 cases.

**Conclusion:**

We found that the prevalence of sickle cell trait is lower as compared to other Arab countries, higher in females, with no significant time trend. The sickle cell test was found to be an accurate, simple and cheap test that could be easily added as a requirement for the pre-marital testing to screen for Sickle Cell Trait carriers.

## Introduction

Sickle syndromes include several inherited diseases that cause red blood cells to sickle in vivo, where the most recognized ones are Sickle Cell Disease (SCD), Sickle Cell Trait (SCT), and Sickle Cell/Thalassemia (S/*β* thalassemia)^1^ Phenotypically, only persons with double recessive genes of sickle cell homozygotes (SS) do manifest disease, while the heterozygotes (AS) are being referred to as Sickle Cell Trait carriers.^2^ It is estimated that each year more than 330,000 children are born worldwide with haemoglobinopathies, 83% of which are sickle cell disorders.^3^

Sickle Cell Disease is a disorder of haemoglobin synthesis that results from the substitution of glutamic acid at the sixth position of its *β*-globin chain by valine (HbS).^4^ It is also known as sickle-cell anemia (SCA) and is characterized by an abnormality in the oxygen-carrying haemoglobin molecule in red blood cells.^5^ It is one of the most common haemoglobinopathies in Africa, the Middle East and India. Sickle cell disease is now found throughout the world, and its incidence has increased in Europe and North America because of the high rate of migration from areas in which the disease is prevalent.^3^,^6^ In 2006, the World Health Organization (WHO) recognized sickle cell disease as a global public health problem.7 According to Diallo et al., Africa is the most highly affected continent with 200,000 newborn affected by sickle cell disease per year.^2^ In the United States, sickle cell disease affects about 72,000 people.^8^ Moreover, Piel et al. expected that the annual global number of newborns with sickle cell disease will increase from 305,800 patients in 2010 to 404,200 patients in 2050 (about one-third increase).^9^ The clinical manifestations of the sickle cell disease are diverse where any organ system may be affected. These signs are commonly divided into vaso-occlusive (where the episodes of pain, acute chest syndrome, splenic infarction, stroke, and avascular necrosis of joints predominate) and hematologic (where severe anemia, leg ulcers, and pulmonary hypertension predominate).^10^,^11^ Early diagnosis and treatment can ameliorate the course of these diseases and improve survival. More specifically, prophylactic penicillin, immunizations, comprehensive care, and parental education about serious sickle cell disease complications has been shown to significantly give a better survival rate and quality of life for sickle cell disease patients.^12^,^13^

Sickle Cell Trait is present in approximately 300 million people worldwide, with the highest prevalence of approximately 30% to 40% in sub-Saharan Africa.^11^

Despite the benign nature of the Sickle Cell Trait, several potentially serious complications have been described with it including, gross hematuria, increased urinary tract infection in women, complications of hyphema, splenic infarction with altitude hypoxia or exercise, and life-threatening complications of exercise.^14^ Hematuria is the most common manifestation of sickle cell trait.^15^ Spontaneous sickling can occur in the renal papilla (normally under low oxygen pressure) in patients with renal papillary necrosis; therefore, 5% of patients with sickle cell trait can suffer episodes of hematuria at some point during their lives.^16^,^17^ Studies in America, England and Jamaica reported that the rates of urinary tract infection are higher for women with sickle cell trait in comparison to racially matched controls.^14^ This finding is well established for asymptomatic bacteriuria of pregnancy, in which the rate is approximately doubled with sickle cell trait.^18^ People with sickle cell trait are more susceptible to complications following treatment of hyphema. The slow flow of relatively hypoxic fluid in the chamber of the eye out of the filtration apparatus is a location in which both polymerization of hemoglobin S and obstruction of flow by rigid erythrocytes is likely. All this can result in glaucoma and secondary hemorrhage.^14^ Splenic Infarction from sickle cell trait is favoured by exercise at high altitude but has occurred with altitude exposure at rest or during exercise at sea level.^11^ The spleen is susceptible to vaso-occlusion related to hemoglobin S polymerization and red cell deformation. When subjects with hemoglobin S are exposed acutely to high altitude hypoxia, the spleen is the organ most consistently injured by microvascular obstruction.^19^ Moreover, there has been growing concern about the possible association of Sickle Cell Trait and exercise-related morbidity and mortality in healthy young college athletes due to the effects of extreme physical exertion, dehydration and relative hypoxia (typically at high altitudes).^1^ The risks of microvascular complications in Sickle Cell Trait carriers in response to exercise are dependent on alterations in blood rheology and vascular adhesion processes. These abnormalities include higher red blood cell rigidity and higher blood viscosity in the sickle cell trait carriers compared with the non-carriers, particularly 24 and 48 hours after exercise.^20^

Pre-marital screening to identify carriers of genetic disorders, especially those with recessively inherited diseases, has been shown to decrease the incidence of inherited Sickle Cell Disease.^21^ This approach is more needed for countries with high prevalence of carriers, such as the Mediterranean region. Various diagnostic tests are available to differentiate between blood samples from normal healthy individuals, Sickle Cell Trait carriers and Sickle Cell Disease patients. Examples of screening tests include sickling test, solubility test, and alkaline haemoglobin electrophoresis on agarose gel.^22^,^23^ On the other hand, high specific diagnostic tests include isoelectric focusing, citrate agar electrophoresis, and high-performance liquid chromatography, as well as the capillary method.^22^,^24^

A study carried out by Khoriaty et al. screened Lebanese neonates for Sickle Cell Disease and other Hemoglobin variants in all public hospitals in Lebanon, between 2010 and 2013. Among newborns, 0.1% were found to have Sickle Cell Disease, and 2.1% were found to have an abnormal Hb variant with HbS being the most common (84.4% corresponding to 1.77% of the studied population) which were distributed in all the regions. The carrier rate of Hb variant varried amongst regions, between 5.7 and 50.2%, being the highest in Northern Lebanon, whereas it is 13.2% (0.27% of the studied population) in Beirut.^25^

This study was conducted to assess the prevalence of Sickle Cell Trait among a sample from a private laboratory (Modern Medical Center/Modern Laboratory) in the Southern Suburb of Beirut, as well as to assess any time trends over a 12-year period, and any differences in gender. Moreover, the importance of screening for Sickle Cell Trait carriers in this population is also highlighted. Another objective was to describe a new “sickling test method” for the screening of Sickle Cell Trait carriers, as well as to assess the correlation of the results with them of the “sodium metabisulfite sickling test”, and to confirm findings with the hemoglobin electrophoresis technique (Sebia Capillarys).

## Methods

### Study design and setting

This research was a retrospective cohort study using data from a private laboratory (Modern Medical Center/Modern Laboratory, established in 1963) in the Southern Suburb of Beirut, Lebanon from 2002 till 2014. Population served by this laboratory includes mainly subjects from a Southern suburb of Beirut, irrespective of their socio-economic status. All patients coming for a complete blood count with differential (CBCD) underwent a free-of-charge sickling test.

### Inclusion and exclusion criteria

Eligible for this study were all subjects referred to our laboratory for CBCD testing, without any exclusion based on age, gender, socioeconomic status or MCV findings. The sample included in our analyses comprised all eligible subjects, where no sampling was applied. Moreover, repetitive results for same patients were excluded.

### Blood testing method

**Figure 1:**
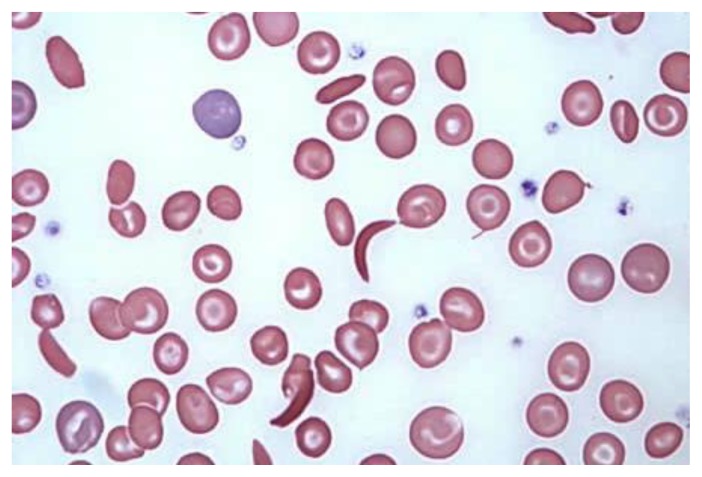
Blood smear of a patient with Sickle Cell Disease.

**Figure 2:**
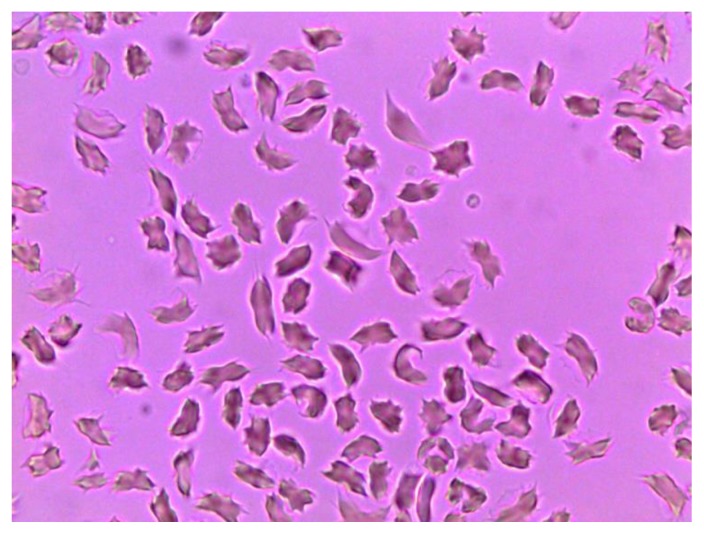
Sickling test of a patient with Sickle Cell Trait.

Blood samples (3 ml) were drawn from patients using EDTA (Ethylenediaminetetraacetic acid) tubes. As a primary screening procedure, the sickling test was carried out for each patient using two methods. In the first method (the new “sickling test method”), one drop of blood was placed on a slide and a cover slide which was sealed using nitrocellulose dissolved in butyl acetate usually found in nail varnish. The slides were incubated at 37°C for 24 hours. As for the second method (“sodium metabisulfite sickling test”), one drop of 2% Sodium metabisulfite was added to a drop of blood between a slide and a cover slide, sealed using the same sealing method and incubated at 37°C for 10 minutes or at room temperature for one hour. For both sets, the slides were inspected under the microscope (Olympus, dry objective 40), and the red blood cells found to be sickling in shape were indicative of a positive sickling test (Figure 1–2). Patients found to be positive were notified of their condition and advised to confirm their results by haemoglobin electrophoresis.

As a confirmatory test, haemoglobin electrophoresis was run on a random sample of 223 subjects who were found to be positive using the sickling test. Another sample of whole blood was drawn from these patients in EDTA tube, and then the red cells were separated from plasma by centrifugation. The Capillarys, an automated capillary electrophoresis by Sebia (Sebia, Surrey, UK), was used in the separation of haemoglobins. The sample was placed in a precise position required by the machine, as well as a red blood cell (RBC) lysis solution at position 27 of the machine and control run simultaneously. Capillarys uses buffer- filled, narrow bore capillaries and detects haemoglobin fractions using UV-visible at 200 nm. Separation occurs according to the electrolyte pH and electroosmotic flow. The test can detect abnormal levels of HbS, the form associated with Sickle Cell Trait and Sickle Cell Disease, as well as other abnormal hemoglobin-related blood disorders.

### Data Collection

Data were collected by extracting the information from the electronic and paper files. For the analysis of the prevalence, information available were a date, age, gender, and MCV. On the other hand, for the confirmatory analysis, information collected included age, gender, and the hematologic parameters such as RBC, Hb (haemoglobin), Hct (hematocrit), MCV (mean corpuscular volume), MCH (mean cell hemoglobin), and MCHC (mean corpuscular hemoglobin concentration). Moreover, information about different types of haemoglobin: HbA1 (haemoglobin, alpha 1), HbA2 (hemoglobin, alpha 2), HbF (fetal hemoglobin) and HbS (hemoglobin S) were included.

### Ethical Considerations

Patients enrolled in our study provided oral consent for the tests to be performed, as well as to be included in the study. The sickling tests were carried out free of charge. Moreover, institutional review board (IRB) of the American University of Science and Technology (AUST) in Lebanon was obtained. Data collected were kept confidential at the principal investigator’s office.

### Statistical analyses

Data entered into a Microsoft Excel spreadsheet and then transferred into the Statistical Package for Social Sciences (SPSS) version 21, which was used for data cleaning, management and analyses. Descriptive analyses were carried out by presenting the number and percent for categorical variables, whereas mean and standard deviation were performed for continuous ones. The prevalence of the sickle cell trait was calculated along with the 95% confidence interval (95% CI). The difference in the prevalence of sickle cell trait, as well as the other hematological parameters between males and females, were assessed by using the students t-test. Statistical significance was identified at 0.05 level.

## Results

**Figure 3:**
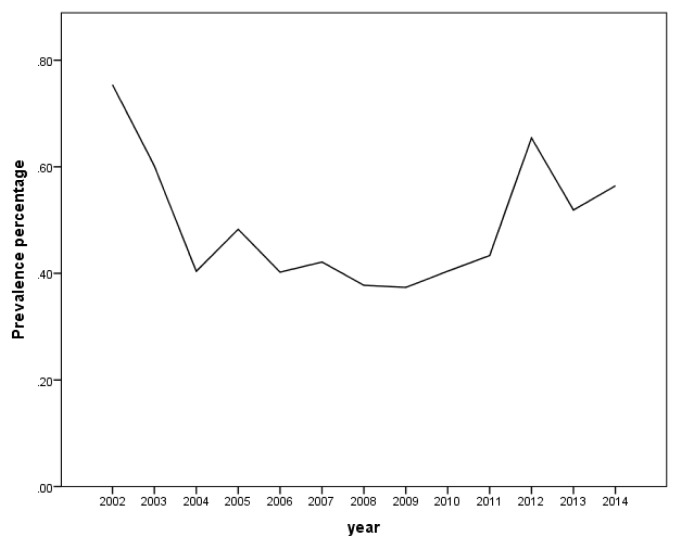
Prevalence of Sickle Cell Trait carriers per year.

**Figure 4:**
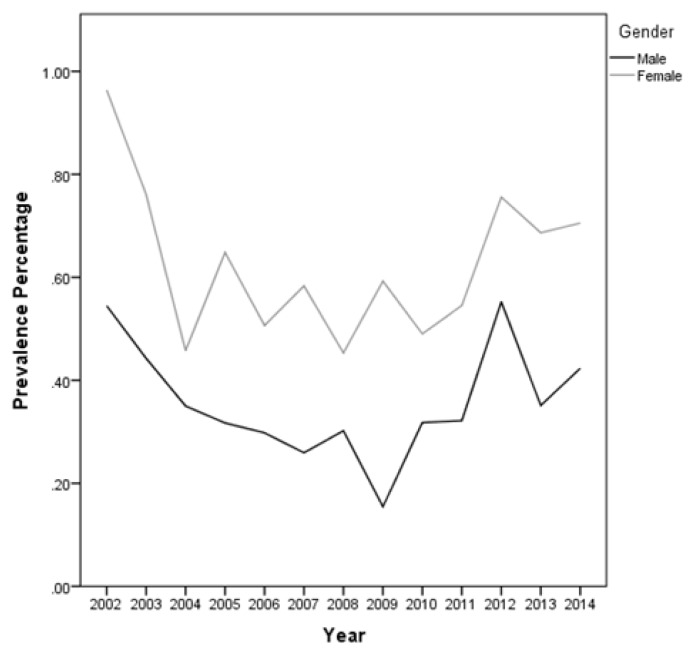
Distribution of Sickle Cell Trait carriers by sex and year.

During the 12-year period between January 2002 and December 2014, a total of 184,105 subjects were screened for sickle cell trait. Over this period, 899 patients were found to be positive for the sickle cell trait, which yielded an overall 12-year sickle cell prevalence of 0.49% (95% CI: 0.46 – 0.52). There was a decrease in prevalence between 2002 from 0.75% (95% CI: 0.61– 0.90) to 0.37% (95% CI: 0.28 – 0.47) in 2009 and then an increase was found from 0.40% (95% CI: 0.30 – 0.51) in 2010 to 0.56% (95% CI: 0.43 – 0.70) in 2014 (Table 1 and Figure 3). Moreover, it was found that 325 males were positive for the sickle cell trait, out of 92,053 subjects yielding a prevalence of 0.35% (95% CI: 0.32 – 0.39), whereas, 574 out of 92,053 were females yielding a prevalence of 0.62% (95% CI: 0.57 – 0.67). Table 2 presents the distribution of sickle cell trait by gender and year. Among males, there was a decrease in prevalence of sickle cell trait between 2002 (0.54%, 95% CI: 0.37 – 0.72) and 2009 (0.15%, 95% CI: 0.07 – 0.24). On the other hand, the sickle cell trait prevalence increased during the period between 2010 and 2014, where the highest prevalence was found to be in 2012 (0.55%, 95% CI: 0.38 – 0.72).. As for females, there was a decrease in prevalence of sickle cell trait from 0.96% in 2002 (95% CI: 0.73 – 1.12) to 0.46% in 2004 (95% CI: 0.30 – 0.61). We did not find any changes in the trends of sickle cell trait prevalence between the years 2005 and 2014. The highest prevalence during this period was found to be in 2012 (0.76%, 95% CI: 0.55 – 0.96), and the lowest was found to be in 2008 (0.45%, 95% CI: 0.30 – 0.61). Worth noting is the finding that the prevalence of sickle cell in each year was lower among males compared to that among females. In some years this difference was statistically significant: p-values of 0.004, 0.01, 0.004, 0.0002, <0.0001, 0.04, 0.009, and 0.04 for the years 2002, 2003, 2005, 2007, 2009, 2011, 2013, and 2014, respectively. These trends are presented in figure 4.

Table 3 presents the average MCV by year and gender. The overall average MCV was found to be 84.3 (±7.6). Stratified by gender, the average MCV for males and females were found to be 84.7(±7.4) and 84.1 (±7.8), respectively, for which the difference was not statistically significant (p-value = 0.24). The average MCV for the different years ranged between 81.8 (±8.3) in 2006 and 86.9 (±9.2) in 2011. The differences in MCV between the genders in the years2002 and 2004 were found to be statistically significant with p-values of 0.01 and 0.02, respectively.

The demographic and hematological characteristics of the 223 cases of positive sickle cell trait which were confirmed by the Hb electrophoresis are summarized in Table 4. The average age of patients was found to be 24.9 years (±15.6) with 40.8% being males. The values of the laboratory results were as follows: RBC = 4.7 million/ul (±0.6), Hb = 12.9 g/dl (±7.6), Hct = 38.1% (±5.1), MCV = 80.6 fL (±9.1), MCH = 26.3 pg (±3.1), MCHC = 32.5 g/dl (±1.1), HbA1 = 57.3% (±7.6), HbA2 = 2.8% (±0.5), HbF = 0.6% (±1.8), and HbS = 38.8% (±5.3).

Finally, Table 5 presents the differences in age and the different hematological parameters between males and females, for the 223 cases with positive HbS screening. Females were older than males with the average age being 26.9 years (±14.3) and 22.1 years (±17.0) for females and males, respectively, p-value = 0.03. A statistically significant difference between males and females was found in the RBC, Hct, and HbA2, the males having highest levels. The p-values were <0.0001 for each of the parameters.

## Discussion

In this retrospective cohort study, that was carried out in a private laboratory in the Sowthern Suburb of Beirut between 2002 and 2014, we assessed the prevalence of Sickle Cell Trait, and time trends over a 12-year period, and any differences in prevalence between the two genders. Moreover, we described a new technique for screening of sickle cell trait carriers, assessing the correlation of the results with those of the known sickling test, and confirmed the findings with the haemoglobin electrophoresis technique (Capillarys). We found the prevalence of Sickle Cell Trait to be 0.49% (95% CI: 0.46–0.52). No major differences in trends were found over the 12-year period. We also found that the prevalence of Sickle Cell Trait was higher in females as compared to males, consistently over the years. The average MCV value among carriers of the sickle cell haemoglobin. Were not diferent from nomal controls. Therefore, the sickling test was found to be a good diagnostic test for Sickle Cell Trait. The prevalence of Sickle Cell Trait in our study was found to be 0.49% (95% CI: 0.46–0.52). Our results were higher than that reported in Khoriaty et al. study in Beirut (0.27% of all Hb variants) but lower than that in all regions of Lebanon (2.1% for all Hb variants and 1.77% for HbS). This difference could be due to the change in the demographics of the Lebanese population, mainly due to emigration and immigration. Another factor that could have affected our results could be due to the change in the ethnic background, as reported by Khoriaty et al..^25^ Also our results were lower than some of the reported prevalence of Sickle Cell Trait internationally. In a study conducted by Ojudu et al., it was reported that in 44 states of the United States for which data were available, of all infants screened for the Sickle Cell Trait in 2010, 1.5% tested positive. These states represent approximately 88% of the U.S. population.^26^ Moreover, a systematic review carried out by Lervolino et al. reported a prevalence ranging from 1.1 % to 9.8% for the Sickle Cell Trait in different Brazilian regions,^27^ and Ballardini et al. reported a 0.7% prevalence of Sickle Cell Trait in a single center in Italy.^28^ On the other hand, Guler et al. assessed the prevalence of sickle cell trait in Konya, the urban area of Turkey, in a study carried out in 2007, where they reported a prevalence of 0.05%, which is lower than that reported in our study.^29^ Compared to other Arab countries, Jastaniah et al. reported in a survey carried out in 2011 that the prevalence of sickle-cell trait in Saudi Arabia varies significantly in different parts of the country and ranges from 2% to 27%.^30^ This rate is higher than that shown in our study. Similarly, some surveys peformed in Middle Eastern countries show that our carrier rate is lower than that reported in many other Arab countries like Bahrain (11.2 – 16.4%),^31^ Iraq (6.5%),^32^ Jordan (4% among females and 6% among males),^33^ Libya (4.5%),^34^ Tunisia (1.9%),^35^ UAE (1.1%)^36^ and Yemen (2.2%).^37^ This difference could be explained in part by the lower rate of consanguineous marriages among the Lebanese population compared to other Arab countries.

The results of the present study showed some gender differences, where the prevalence of Sickle Cell Trait in females (0.62%, 95% CI: 0.57 – 0.67) was higher than that of males (0.35%, 95% CI: 0.32 – 0.39), consistently over the years. Similarly, significant gender differences have been also reported in adults with Sickle Cell Trait carriers. For instance, In a study carried out by Kamble et al. in India, it was reported that male: female ratio was 1.71:1 in HbAS cases.^38^

We did not find any significant differences in the trends of the Sickle Cell Trait over the 12-year period. No studies have been found in the literature reporting on the trends of the Sickle Cell Trait prevalence over time. Nevertheless, our findings indicate a relatively stable prevalence of Sickle Cell Trait over time probably because our studied subjects were born in Lebanon and of Lebanese ancestry. Accordingly, no crucial demographic changes or marital habits (consanguineous) were observed in the past 12 years.

In our study, the overall average MCV was found to be 84.3 fl (±7.6). Similarly, a comparable standard MCV value has been reported among sickle cell trait carriers in France by Tripette et al. in 2009.^39^

On the other hand, in a study done in India, Dangi, et al. in 2010 reported an MCV value of 73.4 fl among the sickle cell trait carriers, which was lower than that reported in our study.^40^ However, this study was performed in an anemic population, and most of these subjects were affected by sickle anemia with beta thalassaemia trait,^40^ and also the iron deficiency cannot be excluded in this setting of patients. We found that the MCV level of the 223 cases of positive sickle cell trait, which were confirmed by the Hb electrophoresis, to be 80.6 (±9.1). MCV levels among the whole sample, as well as the 223 cases, that were tested carrying the Sickle Cell Trait, were within the normal range. Thus, MCV is not useful for screening sickle cell trait, and the microcytosis does not exclude the presence of Sickle Cell Trait as reported by Dangi, [40] and an accurate screening test, like the sickling test, should also be made in patients with microcytosis (microdrepanocytosis according to Silvestroni). 41 Of course, the results should be confirmed by Hb electrophoresis (100% accuracy).

The present study had several limitations. First, it was an observational study without a control group. Another limitation is that the sample was collected from a single center that might not be representative of the whole Lebanese population. As such, the results of the study should be interpreted with these limitations in mind. On the other hand, the study had several strengths, such as the large sample size, the extended period, the prospective data collection, as well as being among the few studies carried out in Lebanon and the region assessing the prevalence of Sickle Cell Trait.

## Conclusion

In this study, we found that the prevalence of Sickle Cell Trait is lower as compared to other Arab countries, higher in females, and with no significant trend over the 12-year period. Moreover, we showed that the MCV, though was not a screening test for Sickle Cell Trait carriers, was useful. In fact, subjects with microcytosis should not be excluded from the selection and could have a contemporaneously thalassemic trait, or sideropenia. On the other hand, the sickle cell test was found to be an accurate, simple and cheap test (It costs approximately two US dollars for both sickling tests) which could be easily added as a requirement for the pre-marital testing to screen for Sickle Cell Trait carriers.

## Figures and Tables

**Table 1:** Distribution of Sickle Cell Trait carriers.

		Total number of sickle cell trait carriers	Base	Prevalence of sickle cell trait carriers	95% confidence interval

**Overall**	**Overall 12 years**	899	184,105	0.49%	0.46% – 0.52%

**By years**	**Year 2002**	108	14,320	0.75%	0.61% – 0.90%
	**Year 2003**	87	14,460	0.60%	0.48% – 0.73%
	**Year 2004**	60	14,855	0.40%	0.30% – 0.51%
	**Year 2005**	70	14,500	0.48%	0.37% – 0.60%
	**Year 2006**	54	13,426	0.40%	0.30% – 0.51%
	**Year 2007**	65	15,425	0.42%	0.32% – 0.52%
	**Year 2008**	55	14,565	0.38%	0.28% – 0.48%
	**Year 2009**	58	15,526	0.37%	0.28% – 0.47%
	**Year 2010**	61	15,096	0.40%	0.30% – 0.51%
	**Year 2011**	62	14,304	0.43%	0.33% – 0.54%
	**Year 2012**	90	13,760	0.65%	0.52% –0.79%
	**Year 2013**	65	12,526	0.52%	0.39% – 0.65%
	**Year 2014**	64	11,342	0.56%	0.43% – 0.70%

By gender*	**Male**	325	92,053	0.35%	0.32% – 0.39%
	**Female**	574	92,053	0.62%	0.57% – 0.67%

* The base was divided by 2 to get the estimate for both males and females and the numbers were rounded up.

**Table 2:** Distribution of Sickle Cell Trait carriers by gender and year.

		Male				Female				

Variables		Total number of sickle cell trait carriers	Base	Prevalence of sickle cell trait carriers	95% confidence interval	Total number of sickle cell trait carriers	Base	Percentage of sickle cell trait carriers	95% confidence interval	p value

**Total sample**		**n=325**				**n=574**				

**Year**	2002	39	7,160	0.54%	0.37% – 0.72%	69	7,160	0.96%	0.73% – 1.12%	0.004
	2003	32	7,230	0.44%	0.29% – 0.60%	55	7,230	0.76%	0.56% – 0.96%	0.01
	2004*	26	7,428	0.35%	0.22% – 0.48%	34	7,428	0.46%	0.30% – 0.61%	0.30
	2005	23	7,250	0.32%	0.19% – 0.45%	47	7,250	0.65%	0.46% – 0.83%	0.004
	2006	20	6,713	0.30%	0.17% – 0.43%	34	6,713	0.51%	0.34% – 0.68%	0.06
	2007*	20	7,713	0.26%	0.15% – 0.37%	45	7,713	0.58%	0.41% – 0.75%	0.002
	2008*	22	7,283	0.30%	0.18% – 0.43%	33	7,283	0.45%	0.30% – 0.61%	0.14
	2009	12	7,763	0.15%	0.07% – 0.24%	46	7,763	0.59%	0.42% – 0.76%	<0.0001
	2010	24	7,548	0.32%	0.19% – 0.45%	37	7,548	0.49%	0.33% – 0.65%	0.09
	2011	23	7,152	0.32%	0.19% – 0.45%	39	7,152	0.55%	0.38% – 0.72%	0.04
	2012	38	6,880	0.55%	0.38% – 0.72%	52	6,880	0.76%	0.55% – 0.96%	0.14
	2013	22	6,263	0.35%	0.21% – 0.50%	43	6,263	0.69%	0.48% – 0.89%	0.009
	2014	24	5,671	0.42%	0.25% – 0.59%	40	5,671	0.71%	0.49% – 0.92%	0.04

* The base was divided by 2 to get the estimate for both males and females and the numbers were rounded up.

**Table 3:** Average of MCV per year and gender.

	MCV (mean ±sd)	Males MCV (mean ± sd)	Females MCV (mean ± sd)	P value

**All years**	84.3 (±7.6)	84.7(±7.4)	84.1 (±7.8)	0.24

**2002**	82.6 (±8.3)	85.4 (±8.0)	81.1 (±8.1)	0.01
**2003**	81.9 (±6.6)	81.2 (±8.4)	82.3 (±5.4)	0.45
**2004**	83.5 (±8.0)	86.3 (±8.5)	81.4 (±7.0)	0.02
**2005**	82.9 (±7.3)	83.5 (±9.2)	82.7 (±6.3)	0.69
**2006**	81.8 (±8.3)	83.6 (±6.4)	80.7 (±9.1)	0.22
**2007**	83.7 (±7.2)	83.7 (±5.6)	83.7 (±7.9)	0.99
**2008**	85.3 (±5.7)	86.3 (±6.6)	84.6 (±5.1)	0.28
**2009**	85.7 (±7.4)	86.8 (±4.4)	85.4 (±8.0)	0.56
**2010**	84.7 (±7.8)	83.6 (±7.9)	85.4 (±7.8)	0.39
**2011**	86.9 (±9.2)	84.7 (±7.9)	88.3 (±9.7)	0.14
**2012**	86.2 (±6.5)	86.1 (±5.9)	86.3 (±7.0)	0.84
**2013**	85.2 (±8.2)	84.2 (±6.0)	85.7 (±9.1)	0.50
**2014**	86.9 (±6.1)	86.7 (±7.5)	87.0 (±5.2)	0.87

**Table 4:** Demographic and hematological parameters for 223 cases with positive HbS screening.

Variables		

Total sample		n=223

**Gender**	Male	91 (40.8%)
	Female	132 (59.2%)

**Age (years)**	Mean (±sd)	24.9 (±15.6)

**RBC (million/ul)**	Mean (±sd)	4.7 (±0.6)

**Hb (g/dl)**	Mean (±sd)	12.9 (±7.6)

**Hct (%)**	Mean (±sd)	38.1 (±5.1)

**MCV (fL)**	Mean (±sd)	80.6 (±9.1)

**MCH (pg)**	Mean (±sd)	26.3 (±3.1)

**MCHC (g/dl)**	Mean (±sd)	32.5 (±1.1)

**HbA1 (%)**	Mean (±sd)	57.3 (±7.6)

**HbA2 (%)**	Mean (±sd)	2.8 (±0.5)

**HbF (%)**	Mean (±sd)	0.6 (±1.8)

**HbS (%) by Hb electrophoresis on capillarys**	Mean (±sd)	38.8 (±5.3)

**Table 5:** Demographic and hematological parameters for 223 cases with positive HbS screening, stratified by gender.

Variables		Male	Female	p value
Total sample		n=91	n=132	
**Age (years)**	Mean (±sd)	22.1 (17.0)	26.9 (14.3)	0.03
**RBC (million/ul)**	Mean (±sd)	5.0 (±0.6)	4.6 (±0.5)	<0.0001
**Hb (g/dl)**	Mean (±sd)	13.1 (±2.0)	12.7 (±9.7)	0.75
**Hct (%)**	Mean (±sd)	40.2 (±5.7)	36.6 (±4.0)	<0.0001
**MCV (fL)**	Mean (±sd)	81.1 (±9.1)	80.2 (±9.0)	0.45
**MCH (pg)**	Mean (±sd)	26.5 (±3.5)	26.2 (±2.8)	0.42
**MCHC (g/dl)**	Mean (±sd)	32.6 (±1.2)	32.5 (±1.0)	0.34
**HbA1 (%)**	Mean (±sd)	56.9 (±8.1)	57.6 (±7.2)	0.50
**HbA2 (%)**	Mean (±sd)	2.9 (±0.5)	2.7 (±0.5)	<0.0001
**HbF (%)**	Mean (±sd)	0.5 (±1.5)	0.6 (±2.1)	0.72
**HbS (%) by Hb electrophoresis on capillarys**	Mean (±sd)	39.2 (±6.8)	38.6 (±3.9)	0.44
